# The Effect of Family Wealth on Physical Function Among Older Adults in Mpumalanga, South Africa: A Causal Network Analysis

**DOI:** 10.3389/ijph.2023.1606072

**Published:** 2023-10-25

**Authors:** Keletso Makofane, Lisa F. Berkman, Mary T. Bassett, Eric J. Tchetgen Tchetgen

**Affiliations:** ^1^ Center for Causal Inference, Department of Biostatistics, Epidemiology and Informatics, University of Pennsylvania, Philadelphia, PA, United States; ^2^ Harvard Center for Population and Development Studies, Harvard University, Cambridge, MA, United States; ^3^ FXB Center for Health and Human Rights, Harvard University, Boston, MA, United States; ^4^ Department of Statistics and Data Science, The Wharton School, University of Pennsylvania, Philadelphia, PA, United States

**Keywords:** causal inference, network spillover, socioeconomic status, epidemiologic study, aging population, physical function, disability

## Abstract

**Objectives:** The aging of the South African population could have profound implications for the independence and overall quality of life of older adults as life expectancy increases. While there is evidence that lifetime socio-economic status shapes risks for later function and disability, it is unclear whether, and how, the wealth of family members shapes these outcomes. We investigated the relationship between outcomes activities of daily living (ADL), grip strength, and gait speed, and the household wealth of non-coresident family members.

**Methods:** Using data from Health and Aging in Africa: A Longitudinal Study of an INDEPTH Community in South Africa (HAALSI) and the Agincourt Health and Demographic Surveillance System (AHDSS), we examined the relationship between physical function and household and family wealth in the 13 preceding years. HAALSI is a cohort of 5,059 adults who were 40 years or older at baseline in 2014. Using auto-g-computation—a recently proposed statistical approach to quantify causal effects in the context of a network of interconnected units—we estimated the effect of own and family wealth on the outcomes of interest.

**Results:** We found no evidence of effects of family wealth on physical function and disability.

**Conclusion:** Further research is needed to assess the effect of family wealth in early life on physical function and disability outcomes.

## Introduction

A demographic transition is underway in South Africa. Between 2020 and 2030, the proportion of the population that is over 65 will grow by one fifth—from 5.5% in 2020 to 6.7% in 2030 [1]. If not offset by improvements in healthy aging, this transition could have profound implications for the independence and overall quality of life of older adults [2]. To anticipate how these changes could be patterned in South Africa in the future, we investigated the relationship between socioeconomic status and outcomes related to disability.

Over the life course, social status arrays a multitude of exposures which are “embodied” by individuals, shaping later health outcomes [3–5]. Prior studies have established a robust positive association between higher socioeconomic status and performance-based measures of physical function [6–10] and a negative association with self-reported limitations in basic and instrumental activities of daily living [11–14]. There is consistent evidence that lower childhood socioeconomic status is associated with lower physical function in later life [14–16], and mixed evidence that these effects persist even after accounting for adult socioeconomic status [17, 18]. These findings are based largely on studies that were conducted in high income countries, though there is a growing body of evidence from low- and middle-income countries including South Africa [19, 20].

While it is clear that individuals’ access to material resources shapes the risk of disability, it is not well understood whether and how the resources held by family members shape these outcomes. This is a crucial gap. In South Africa, households are embedded in local kinship-based networks of support, exchanging resources including labor and food with one another [21]. Under the assumption that the wealth of a particular household is a determinant of its members’ ability to provide resources to members of other households, the level of wealth held by family members could be an important determinant of the physical function of the individual.

Guided by the Disablement Process [22, 23], a conceptual model that posits a causal pathway moving from pathology to impairment to functional limitation and to disability, we investigated the relationship between socioeconomic status and Activities of Daily Living (ADL), grip strength, and gait speed. The ADL scale is widely used to assess the severity of limitations among older and chronically ill patients that hinder the completion of tasks thought to be habitual and universally performed: bathing, dressing, going to the toilet, transferring, continence, and feeding [24]. We consider it a measure of disability. We consider hand grip strength to be a measure of impairment, assessing overall losses in muscle mass and strength [25], and gait speed to be measure of functional limitation, assessing the ability to perform the action of walking. Understanding how functional limitations and disability are shaped by the wealth of family members could shape welfare policy by, for instance, identifying the need for additional support among poor households which are connected only to other poor households (compared with poor households which are connected to wealthy ones).

Using data from Health and Aging in Africa: A Longitudinal Study of an INDEPTH Community in South Africa (HAALSI) and the Agincourt Health and Demographic Surveillance System (AHDSS), we examined the relationship between physical function and household wealth in the 13 preceding years. We quantified the effect of a one-standard-deviation increase in household wealth on average physical function using regression analysis. We establish a causal interpretation for these regression results drawing on auto-g-computation—a recently proposed statistical approach to quantify spillover causal effects on a network of interconnected units [26]. This approach allows us to decompose the overall effect of household wealth on disability and physical function into a causal component attributable to the change in each individual’s own household resources, and a component attributable to the change in the household resources of non-coresident family members. We present estimates of these effects, along with the assumptions under which they may be interpreted as causal.

## Methods

### Data and Study Setting

Covering a 420 $km^{2}$ region of Mpumalanga Province, South Africa, Agincourt Health and Demographic Surveillance System (AHDSS) has conducted an annual survey of households, collecting information on births, deaths, migrations, and family relationships since 1992. In addition, AHDSS fieldworkers collected information on household wealth once every 2 years beginning in 2001 and annually since 2013 [27, 28]. The Health and Aging in Africa: A Longitudinal Study of an INDEPTH Community in South Africa (HAALSI) is a longitudinal cohort or older adults nested in the AHDSS. The HAALSI cohort is comprised of 5,059 adults who were sampled from residents of AHDSS who were over 40 years of age in 2014 [29].

We used AHDSS data on household wealth as the main exposure and HAALSI data on disability and physical function as the main outcome. Since this study used secondary data, IRB approvals were not required.

### Social Network

To quantify the resources held in family networks, we constructed a sociocentric family network among all individuals in the AHDSS. In this network, nodes represent individuals and ties represent their relationships with first- or second-degree relatives. First-degree relatives are defined as parents, children, and conjugal partners, and second-degree relatives are the first-degree relatives of first-degree relatives. For a given individual, we use the term ‘family members’ to mean the group consisting of first- and second-degree relatives.

We used a sub-network consisting only of members of the HAALSI cohort along with their family members, regardless of whether the latter were themselves in the HAALSI cohort or not. We call this the HAALSI community network (HCNet).

### Measures

#### Household and Network Wealth

**FIGURE 1:**
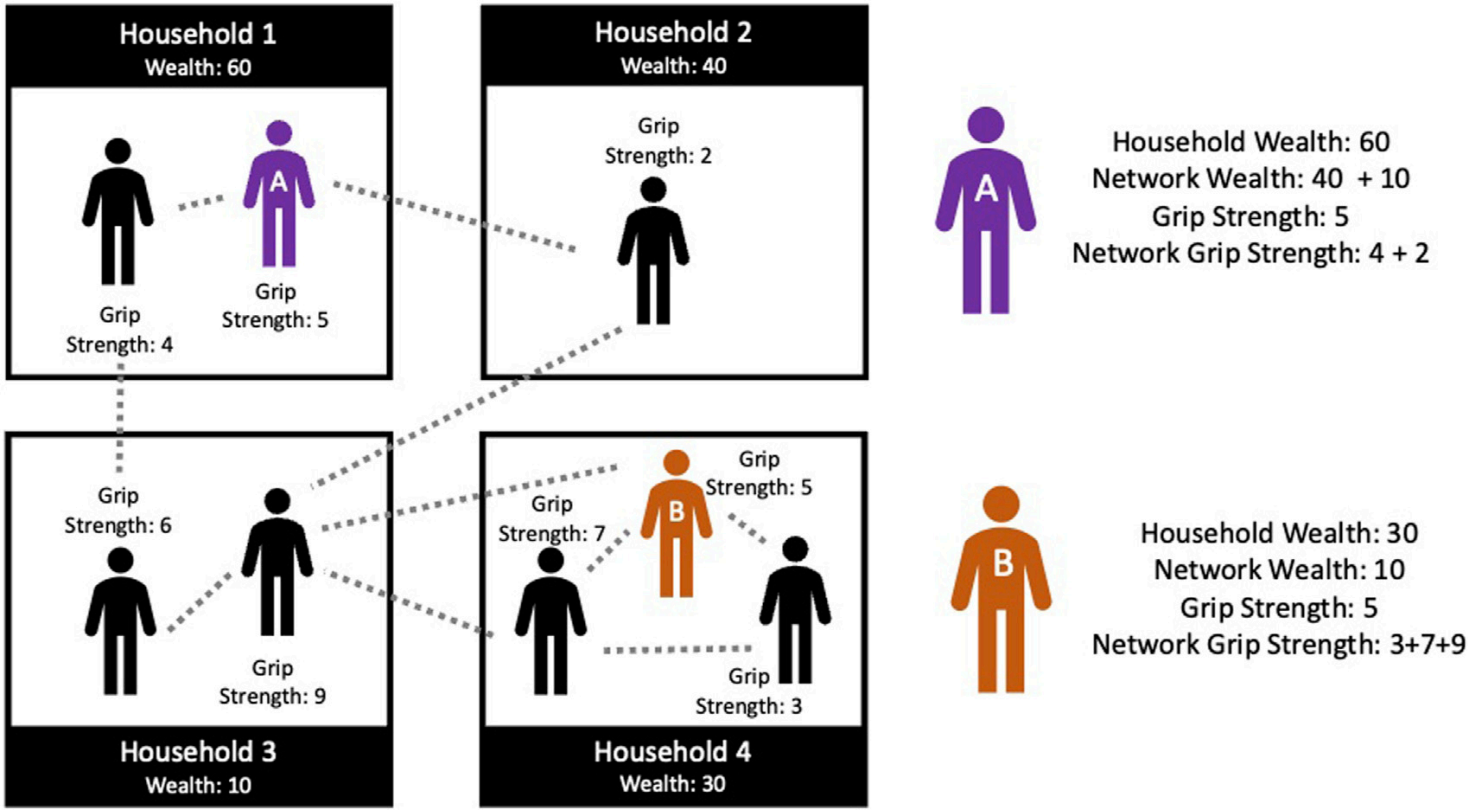
Agincourt Health and Demographic Surveillance System Network Schematic. Dotted lines indicate family relationships. Boxes indicate households. Each individual has an individual Grip Strength score and each household has a Household Wealth value. Network Wealth is the sum of Household Wealth values for connected households. Network Grip Strength is the sum of Grip Strength scores among connected individuals. (Health and Aging in Africa: A Longitudinal Study in South Africa, Mpumalanga, South Africa, 2001 to 2014).

Household wealth was assessed using a Demographic and Health Survey (DHS) asset index that incorporates information on household infrastructure and goods [20, 30]. Measurements of household wealth were made every 2 years from 2001 to 2013. For each year in this period, each individual in the HCNet is associated with the measure of household wealth that was recorded for her household. Network wealth was calculated as the sum of household wealth among the households of family members (See Figure 1). Where two or more family members lived in the same household, that household’s wealth contributed only once to network wealth.

#### Individual and Network Physical Functioning

In HAALSI, individual grip strength was measured using a Smedley digital hand dynamometer, taking two measurements per hand. Following [20], we used the average of the grip strength measures on the participant’s self-reported dominant hand. For participants who reported being ambidextrous, we took the average of the two highest measures regardless of which hand they were measured on. Measures above 75 kg were treated as out of range and therefore missing. An individual’s network grip strength was measured as the sum of grip strength values among her family members who were over 40 years of age in 2014 .

#### Individual and Network Gait Speed

Individual gait speed was measured among HAALSI participants using a timed walk. Interviewers marked a length of 2.5 meters on an obstacle-free floor. The respondent was asked to walk from one end to the other, and she was timed. The respondent was then asked to turn around and return to the point of origin while being timed. Gait speed was calculated by dividing 5 by the sum of the times (in seconds). Gait speeds below 0.2 m/s or above 2 m/s were treated as out of range and therefore missing. Network gait speed was measured as the sum of gait speed values among family members who were over 40 years of age in 2014 .

#### Individual and Network Activities of Daily Living

Limitations in activities of daily living (ADL) was measured in HAALSI using a set of questions asking whether the respondent is unable, or finds it difficult, to bathe, eat, get out of bed, toilet, or walk across the room unaided. Individual ADL is equal to 1 if the individual had at least one limitation and 0 otherwise. Network ADL is the sum of ADL values among each individual’s family members who were above 40 years of age in 2014 .

### Statistical Analysis

We conducted descriptive analysis for the above measures, as well as covariates formal education, employment, receipt of pension income, children, and marital status at HAALSI baseline. We also used these variables in the data imputation model. We included age and gender as potential confounders of the relationships between wealth measures and physical function measures. To help explain a surprising finding below, we conducted further analysis comparing time spent walking across individuals in different wealth quartiles. We present an abbreviated discussion of statistical and causal inference methods here. Details can be found in Supplementary Material.

#### Statistical Model

We assume the outcome data arose from a conditional Markov Random Field [26, 31] defined by the assumption that one individual’s outcome is independent of any other individual’s observations if a) the pair of individuals are not each other’s family members b) we condition on the exposures and covariates of the first individual and her family members, and on the outcomes of family members.

We further assume that the conditional mean of physical function is described by the following model:

$$E\left[Y_{i,2014}^{ind}|L=l,A=a,Y=y\right]=\beta_{0,t}+\beta_{1,t}a_{i,t}^{hh}+\beta_{2,t}l_{i}^{ind}+\beta_{3,t}a_{i,t}^{net}+\phi_{2014}y_{i,2014}^{net}$$

where for individual i during year t , $Y_{i,2014}^{ind}$ is the value of the individual outcome, $y_{i,2014}^{net}$ is the value of the network outcome, $a_{i,t}^{hh}$ is household wealth, $a_{i,t}^{net}$ is network wealth, and $l_{i}^{ind}$ is an individual-level covariate value. Using linear regression, we estimated this model separately for each year t and each of the three physical function outcomes under investigation.

Though we only show one covariate, in analyses, this model included time-invariant covariates age (40–50; 51–60; 61–70; >70) and gender (male; female) as measured in AHDSS since these potentially determine household wealth and physical function.

#### Causal Estimands

Using auto-g-computation, we establish causal interpretation for the parameters of the conditional mean model shown above [26]. Combining estimated parameter estimates from the model with information on network structure, household membership, and age among AHDSS residents, we calculated the average direct effect (ADE), average spillover effect (ASE), and average total effect (ATE) of household wealth on disability and physical function.

For a given individual, we define the spillover effect as the change in outcome that results from holding the individual’s household wealth fixed while increasing wealth for every other individual by one standard deviation. ASE is then the quantity we estimate by computing the spillover effect separately for each individual in the network and then taking an average over the network. Conversely, we define the direct effect as the change in outcome that results from increasing an individual’s household wealth by one standard deviation, holding fixed the household wealth of all others. ADE is the average of this quantity. ATE is the on-average change in outcome that would result from a simultaneous one standard deviation increase in wealth for every household. ATE is the sum of ADE and ASE.

#### Identification Assumptions

ADE, ASE, and ATE are identified under the assumption that there is no unaccounted-for confounding. We included age and gender as potential confounders in the conditional mean model. This is consistent with past studies on the impact of socioeconomic status on health as assessed over long time horizons [8, 15, 32–34].

Unconfoundedness in the network setting also implies that there are no unmeasured common causes of the physical function measures taken on a pair of family members. If this were untrue, physical function outcomes would be correlated among family members—a condition we empirically test. Finally, we assume that the functional form we chose for the conditional mean model is correct.

#### Estimation

Parameters of the conditional mean model were estimated using general estimating equations with robust standard errors. We constructed Wald confidence intervals for each of these. Since ADE, ASE, and ATE are deterministic functions of the coefficients, we estimated them by plugging-in estimated values of the coefficients into the function. Based on the asymptotic distribution of the regression coefficients, we constructed confidence intervals for these causal estimates using the parametric bootstrap.

Estimating the parameters of the conditional mean models was complicated by the fact that the observations belonging to a pair of individuals who are connected in the family network are possibly correlated with each other. This is true when $\phi_{2014,q}\neq 0$ . In this case, proceeding as if the observations were independent may lead to biased estimates. To overcome this challenge, we used the coding estimator described in [26], which requires that the conditional mean model be fitted using a sub-set of the data such that no pair of individuals in the sub-set is connected in the family network. Further details are shown in Supplementary Material.

#### Missing Data

Some individuals were missing data on household wealth, inducing missingness in network wealth. In addition, physical function was only measured among HAALSI participants and not among all AHDSS residents. This meant that among HAALSI participants, network physical function measures are not possible to compute directly. If we attempted to compute network physical function by summing over only the valid values of physical function among each respondent’s direct ties, they would be right-censored. To account for missingness, we conducted two separate sets of analyses. The main analysis, which we present here, is based on multiple imputation using chained equations. This analysis is predicated on the assumption that missingness depends only on observed variables [35]. The secondary analysis, reported in Supplementary Material, dropped entries that were missing the outcome value or mean-imputed household wealth. Results from the latter analysis are unbiased under the stronger assumption that data are missing completely at random [35]. Details about imputation are shown in Supplementary Material.

## Results

### Descriptive Analysis

At baseline, a quarter of HAALSI respondents were over 70 years of age and half were over 60 years of age (Table 1). Slightly more than half of the respondents were women, over half had some formal education and a small minority were employed, about a third received pension income, about half were married and/or living with a romantic partner, and the vast majority had children. Respondents whose household wealth was above the median tended to have higher formal education and employment, were more likely to receive pension income and were more likely to have children. They also tended to have higher wealth embedded in their family network. Wealthier members of the HAALSI cohort had higher grip strength, lower gait speed, and fewer limitations in activities of daily living than poorer members.

**TABLE 1:** Descriptive Statistics. (Health and Aging in Africa: A Longitudinal Study in South Africa, Mpumalanga, South Africa, 2001 to 2014).

	Above median wealth (N = 2,374)	Below median wealth (N = 2,378)	Overall (N = 4,752)
Age			
40–49	463 (19.5%)	491 (20.6%)	954 (20.1%)
50–59	637 (26.8%)	656 (27.6%)	1,293 (27.2%)
60–69	680 (28.6%)	529 (22.2%)	1,209 (25.4%)
70+	593 (25.0%)	702 (29.5%)	1,295 (27.3%)
Missing	1 (0.0%)	0 (0%)	1 (0.0%)
Gender			
Male	1,098 (46.3%)	1,084 (45.6%)	2,182 (45.9%)
Female	1,276 (53.7%)	1,294 (54.4%)	2,570 (54.1%)
Any Formal Education			
Yes	1,533 (64.6%)	1,019 (42.9%)	2,552 (53.7%)
No	832 (35.0%)	1,353 (56.9%)	2,185 (46.0%)
Missing	9 (0.4%)	6 (0.3%)	15 (0.3%)
Employment			
Yes	403 (17.0%)	314 (13.2%)	717 (15.1%)
No	1964 (82.7%)	2058 (86.5%)	4,022 (84.6%)
Missing	7 (0.3%)	6 (0.3%)	13 (0.3%)
Receives Pension Income			
Yes	859 (36.2%)	826 (34.7%)	1,685 (35.5%)
No	1,515 (63.8%)	1,552 (65.3%)	3,067 (64.5%)
Has Children			
Yes	2,283 (96.2%)	2,183 (91.8%)	4,466 (94.0%)
No	89 (3.7%)	194 (8.2%)	283 (6.0%)
Missing	2 (0.1%)	1 (0.0%)	3 (0.1%)
Married			
Yes	1,438 (60.6%)	1,014 (42.6%)	2,452 (51.6%)
No	933 (39.3%)	1,363 (57.3%)	2,296 (48.3%)
Missing	3 (0.1%)	1 (0.0%)	4 (0.1%)
Grip Strength			
Mean (SD)	24.3 (8.74)	23.2 (8.64)	23.7 (8.71)
Median [Min, Max]	23.1 [0, 73.6]	22.3 [0, 74.6]	22.7 [0, 74.6]
Missing	218 (9.2%)	230 (9.7%)	448 (9.4%)
Gait Speed			
Mean (SD)	0.672 (0.243)	0.689 (0.275)	0.680 (0.259)
Median [Min, Max]	0.625 [0.200, 1.67]	0.625 [0.200, 1.67]	0.625 [0.200, 1.67]
Missing	178 (7.5%)	193 (8.1%)	371 (7.8%)
Any ADL			
Yes	176 (7.4%)	237 (10.0%)	413 (8.7%)
No	2,191 (92.3%)	2,141 (90.0%)	4,332 (91.2%)
Missing	7 (0.3%)	0 (0%)	7 (0.1%)
Network Wealth			
Mean (SD)	12.2 (6.26)	10.1 (6.79)	11.1 (6.62)
Median [Min, Max]	11.4 [0, 41.0]	9.96 [0, 42.2]	10.7 [0, 42.2]
Missing	720 (30.3%)	603 (25.4%)	1,323 (27.8%)

### Conditional Mean Model

**FIGURE 2:**
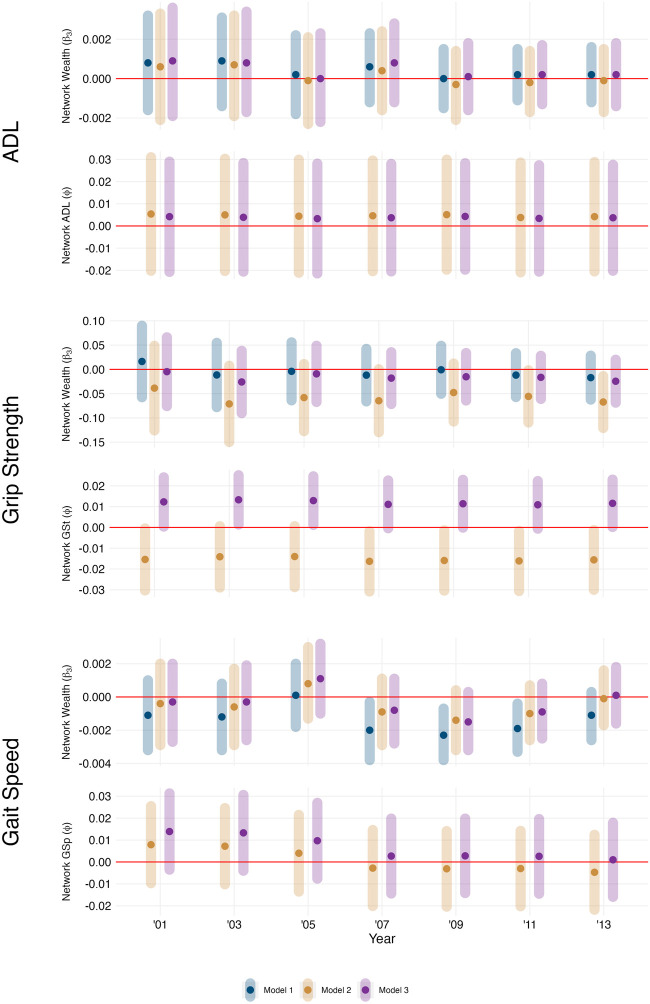
Conditional Mean Model Results for Activities of Daily Living (ADL), Grip Strength (GSt), and Gait Speed (GSp). Model 1 includes Network Wealth and Household Wealth as predictors. Model 2 includes these as well as Network ADL as predictors. Model 3 includes these as predictors and adjusts age and gender as potential confounders. The top row shows the point estimate and confidence interval for network wealth ( $\beta_{3,t}$ ), and the second bottom shows the coefficient for the network value of the physical function outcome ( $\phi_{2014}$ ). (Health and Aging in Africa: A Longitudinal Study in South Africa, Mpumalanga, South Africa, 2001 to 2014).

In Figure 2, we show results from the conditional mean models for ADL, grip strength, and gait speed, respectively. Since we did not reject the hypothesis that ϕ=0 for ADL or gait speed, we concluded that for these outcome measures, observations were conditionally independent. i.e., Knowing about one individual’s ADL or gait speed does not provide any information about the ADL or gait speed of individuals directly connected to him after adjusting for individual and network exposures and covariates. As a result, for these two outcomes, we fitted regression models using all available data. By contrast, there was evidence of positive conditional network dependence of grip strength among directly connected individuals, so we used the coding estimator described above, which required the use of a subset of data for estimation.

### Causal Estimates

**FIGURE 3:**
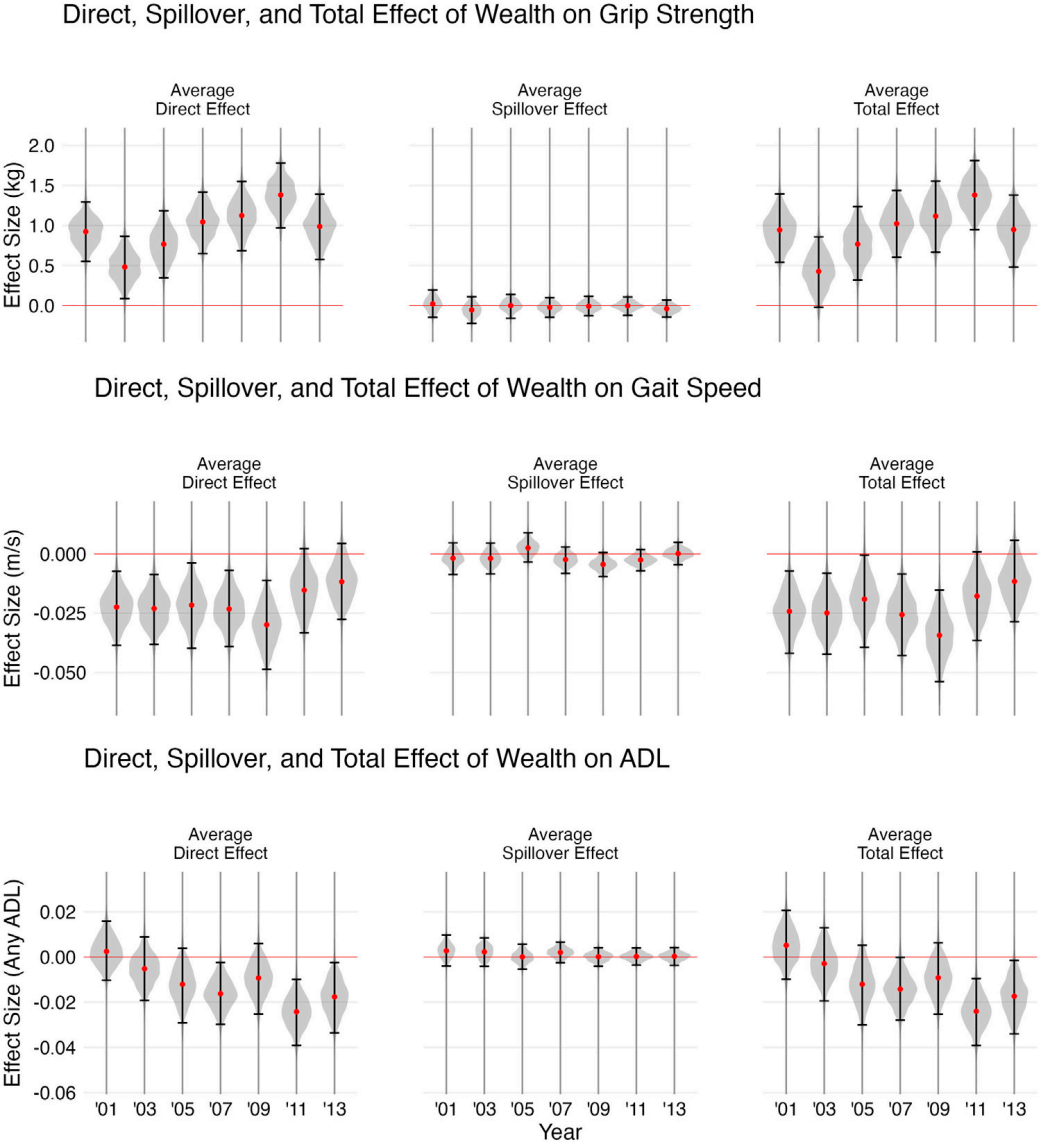
Causal Estimates of the Effect of Wealth on Activities of Daily Living (ADL), Grip Strength, and Gait Speed. The first column shows the point estimate and confidence interval for Average Direct Effect (ADE), the second column shows results for Average Spillover Effect (ASE), and the final column shows results for Average Total Effect (ATE). (Health and Aging in Africa: A Longitudinal Study in South Africa, Mpumalanga, South Africa, 2001 to 2014).

Since for gait speed and for ADL, network wealth and network physical function were not associated with the outcome, as shown in Figure 3, the average spillover effect was 0 for both these outcomes. There was some statistical evidence of a positive average direct effect of household wealth on grip strength in the years 2001 (0.923 95% CI: 0.551–1.294), 2003 (0.481 95% CI: 0.087–0.864), 2005 (0.767 95% CI: 0.346–1.184), 2007 (1.044 95% CI: 0.649–1.417), 2009 (1.124 95% CI: 0.684–1.55), 2011 (1.382 95% CI: 0.969–1.779), and 2013 (0.987 95% CI: 0.574–1.391), though the average spillover effect of household wealth on grip strength was 0 across all years for this outcome as well. There was evidence of a negative direct effect of wealth on ADL in the years 2007 (−0.016 95% CI: −0.03 to −0.002), 2011 (−0.024 95% CI: −0.039 to −0.01), and 2013 (−0.018 95% CI: −0.034 to −0.003).

**FIGURE 4:**
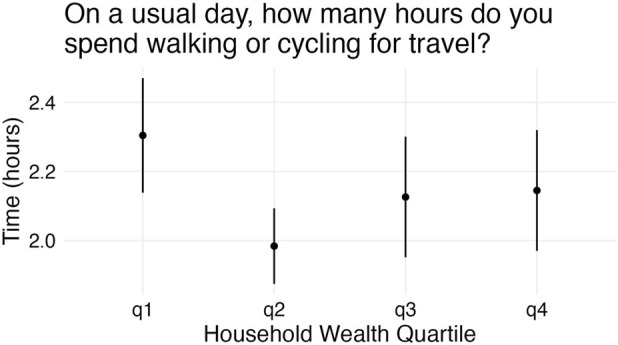
Time spent walking by Household Wealth. (Health and Aging in Africa: A Longitudinal Study in South Africa, Mpumalanga, South Africa, 2001 to 2014).

Surprisingly, there was evidence of a negative direct effect of wealth on gait speed in 2001 (−0.022 95% CI: −0.038 to −0.007), 2003 (−0.023 95% CI: −0.038 to −0.009), 2005 (−0.022 95% CI: −0.04 to −0.004), 2007 (−0.023 95% CI: −0.039 to −0.007), and 2009 (−0.03 95% CI: −0.049 to −0.011). One possible explanation for this is that wealth shapes the amount of time spent walking. HAALSI respondents in the lowest quartile of household wealth walked for 2.3 h (95% CI: 2.14–2.47) per day while those in the second lowest quartile walked for 2 h (1.87–2.09) and those in the highest quartile walked for 2.1 h (1.97–2.32) (See Figure 4).

## Discussion

Overall, we found no evidence of spillover effects of household wealth on measures of physical function or disability. We found evidence of a direct effect of household wealth on slower gait speed and a direct effect of household wealth on higher grip strength. Finally, we found weak evidence of a direct negative effect of wealth on limitations in ADL.

These measures offer different insights about the disablement process. We consider ADL a limited measure of disability, assessing the perceived capability of individuals to accomplish tasks demanded by their social environment without directly measuring those demands [23]. We consider hand grip strength to be a measure of impairment, assessing overall losses in muscle mass and strength [25] and gait speed to be measure of functional limitation, assessing the ability to perform the action of walking. The latter two measures are “objective” and not based on self-report. Unlike ADL, they detect relatively small changes in function and are not susceptible to the systematic biases associated with self-report, though they might be susceptible to other kinds of biases [4].

For grip strength and ADL, our results are broadly consistent with the extant literature on functional limitations and disability among older adults. Socio-economic status has consistently found to be positively correlated with performance-based measures of physical function [6–10] and negatively associated with variables based on self-reported limitations in basic and instrumental activities of daily living [11–14]. That household wealth is negatively associated with gait speed is inconsistent with similar studies globally, but consistent with earlier work using the HAALSI dataset; [20] found that those in the highest quintile of household wealth had lower gait speed than those in the lowest quintile.

Our finding of null spillover effects of socioeconomic status echoes a result from a comparable study. Using the China Health and Retirement Longitudinal Study data which, like HAALSI, is a Health and Retirement Study (HRS) sister study, [36] found that “family economic support” — financial support by parents, children, or siblings—was not associated with health outcomes, including ADL, among older adults. It should be noted, however, that family economic support was not quantified as in our study; it was operationalized as a dichotomous variable indicating whether there was any family support or not.

There may be contextual reasons for the negative association between wealth and gait speed. Cross-country comparisons show that not only are population levels of impairment, limitation, and disability variable across settings [13, 20, 37, 38], the quantitative relationships among socioeconomic status, physical function, and self-reported limitations are context-dependent as well ([39]; [40–42]). In particular, in Agincourt, people of lower socio-economic status do more walking than those of higher status, possibly accounting for their faster walk speed. There is a body of research suggesting that increased physical activity plays a role in the prevention of limitations in physical function [43, 44].

Alternatively, this negative association could be a result of sample selection bias. Higher wealth likely increased the probability of surviving until enrollment into HAALSI through mechanisms other than gait speed, and higher gait speed might have also been positively associated with the probability of being in the HAALSI cohort. This kind of bias would induce a negative association between gait speed and wealth.

It is possible that there are spillover effects of wealth on physical function and disability that we failed to detect in this study. Our earliest measurements of wealth were in 2001—only 13 years prior to the measurement of the health outcomes. For the youngest members of HAALSI, wealth was measured beginning in their early 30s. For the oldest members of HAALSI, wealth was measured from their late 50s. These measures might miss an earlier critical window during which shared resources are more important, etiologically. There is robust evidence that early life conditions shape mid-life physical function. Birth weight, pre-pubertal height gain, pubertal growth, infant motor development all predict mid-life grip strength [45].

It is not possible to rule out reverse causation: it might be the case that prior ADL limitations, low gait speed and weak hand grip lowered the ability of HAALSI participants to generate income for their households due to unemployment [46], leading to lower wealth. It might also be that these effects were transmitted across family ties. Agincourt, however, has a chronically high unemployment rate, ranging from 63% as measured in the 2001 census to 52% as measured in the 2011 census [47]. As a result, the share of household income that is comprised of government transfers, including the state disability grant, is high. In 2019, Statistics South Africa reported this figure to be 54% across Mpumalanga Province, with a further 22% of income gained from remittances [48]. It is not clear whether functional limitations would decrease income via wages or increase income via grants and remittances, so it is difficult to anticipate the existence and direction of bias due to reverse causation.

Finally, physical function outcomes were likely measured with error, potentially attenuating the strength of the relationships between (network) wealth and the outcomes. Furthermore, our measure of socioeconomic status might be relatively insensitive to social gradients as they affect physical function, and therefore insensitive to networked social gradients. A prior study using HAALSI data showed steeper gradients in health outcomes when using a consumption-based measure of socioeconomic status rather than the wealth-based measure we used here [49]. Given the high unemployment and large number of households that receive government transfers in Agincourt, it is possible that using government transfers as a measure of socioeconomic status would show stronger social gradients than we found. It is also possible that understanding which relationships are more likely to transmit resources than others would allow for the detection of stronger social gradients. Future studies should investigate spillover using consumption-based measures of socio-economic status as well as measures based on government transfers along with the wealth-based measures we have used here and measures of the nature and closeness of relationships among family members.

Our study provides evidence that in adulthood, impairment, physical limitation and disability are shaped by one’s own household wealth but not necessarily the wealth of family members in other households. These results suggest that knowing about the socio-economic status of family members will not help us to predict the burden of disability among the aging South African population. Further research is needed to assess the effect of wealth and family wealth in early life on these same outcomes, and if those effects are significant, to assess the different causal pathways that connect exposure and outcome. To enable this work, it is crucial to cultivate network datasets, possibly combining data from epidemiologic studies with data that are passively collected from social media and mobile devices.
