# Supplementary material for: The Effect of Family Wealth on Physical Function Among Older Adults in Mpumalanga, South Africa: A Causal Network Analysis
Source: Int J Public Health. 2023 Oct 25;68:1606072. doi: 10.3389/ijph.2023.1606072 (PMC10630774; doi:10.3389/ijph.2023.1606072)
Supplement: Supplementary file 1 [file DataSheet1.DOCX]

Networked wealth and baseline physical function among HAALSI cohort members

Supplementary Materials

# Introduction

In these supplementary materials, we describe the identification strategy and estimation procedures for “Networked wealth and baseline physical function among HAALSI cohort members”, reporting a full set of results based on all analyses conducted.

# Notation

A summary of notation can be found in [Box 1](#thm-symbols). We define a graph $\mathcal{G}$ with $n$ nodes that represent individuals and ties that represent family relationships. Since individuals are nested in households, we use $\mathcal{G}$ along with information on household membership to induce a network of households $\mathcal{H}$ of size $h$ as follows: Each node in $\mathcal{H}$ (i.e. each household) represents a sub-set of nodes in $\mathcal{G}$ (i.e. a set of individuals). There is a tie between a pair of households in $\mathcal{H}$ if and only if there is at least one individual in the first household who is related to an individual in the second household, according to $\mathcal{G}$.

Formally, we define graph $\mathcal{G}\equiv\left( N^{\mathcal{G}},E^{\mathcal{G}} \right)$, an ordered pair consisting of a set of nodes $N^{\mathcal{G}}$ and a set of ties $E^{\mathcal{G}}\equiv\{\{i,j\}:i,j\in N^{\mathcal{G}}\text{ and }i\text{ is connected to }j\text{ in }\mathcal{G}\}$. Graph $\mathcal{G}$ is associated with adjacency matrix $M^{\mathcal{G}}$ with entries

$$m_{ij}^{\mathcal{G}}=\left\{ \begin{matrix} 1 & \text{if }\{i,j\}\in E^{\mathcal{G}},i\neq j \\ 0 & \text{otherwise} \end{matrix} \right..$$

We further define graph $\mathcal{H}\equiv\left( N^{\mathcal{H}},E^{\mathcal{H}} \right)$ by coarsening graph $\mathcal{G}$. $N^{\mathcal{H}}$ is a partition of $N^{\mathcal{G}}$ based on household membership, i.e. for $I,J\in N^{\mathcal{H}}$,

$$\begin{matrix} I,J\subset N^{\mathcal{G}} \\ I,J\neq\emptyset, \\ I\cap J=\emptyset\\ \underset{S\in N^{\mathcal{H}}}{\bigcup}S=N^{\mathcal{G}} \end{matrix}.$$

Then $E^{\mathcal{H}}$ is defined as set $\{\{I,J\}\in N^{\mathcal{H}}\times N^{\mathcal{H}}:\exists\{i,j\}\in E^{\mathcal{G}}\text{ where }i\in I,j\in J \}$. Graph $\mathcal{H}$ is associated with adjacency matrix $M^{\mathcal{H}}$ defined analogously to $M^{\mathcal{G}}$.

We define the household roster matrix $M^{\mathcal{H}\mapsto\mathcal{G}}$ – an $n\times h$ matrix with entries

$$m_{ij}^{\mathcal{H}\mapsto\mathcal{G}}=\left\{ \begin{matrix} 1 & \text{ when }i\in j\text{ for }i\in N^{\mathcal{G}},j\in N^{\mathcal{H}} \\ 0 & \text{ otherwise } \end{matrix} \right..$$

We define $N_{i}\subset N^{\mathcal{G}}$ as the set containing individual $i$ as well as the individuals connected to $i$ in the graph $\mathcal{G}$, $H_{i}\in N^{\mathcal{H}}$ as individual $i$’s household, and $\Gamma_{i}\subset N^{\mathcal{H}}$ as a set containing $i$’s household as well as all households connected to $i$’s household. $\mathbf{1}_{n}$ is a $n\times1$ vector of $1$’s and $\mathbf{I}_{n}$ is the $n\times n$ identity matrix.

Finally, we define a $n\times1$ vector of dichotomous potential outcomes $\mathbf{Y}\left( \mathbf{a} \right)$ under $h\times1$ vector $\mathbf{a}$. The $i^{th}$ entry of vector $\mathbf{Y}\left( \mathbf{a} \right)$ represents the physical function score individual $i$ would obtain if households were forced to have wealth as dictated by $\mathbf{a}$. The $i^{th}$ entry of $\mathbf{a}$ represents the value of the wealth index imposed on household $i$.

**Box 1 (Definitions)**

$$\begin{matrix} \mathcal{G}\equiv\left( N^{\mathcal{G}},E^{\mathcal{G}} \right) & \text{Graph representing individuals and family relationships} \\ N^{\mathcal{G}} & \text{Nodes (individuals) of graph }\mathcal{G} \\ E^{\mathcal{G}} & \text{Ties (family relationships) of graph }\mathcal{G} \\ n & \text{Number of nodes in graph }\mathcal{G} \\ M^{\mathcal{G}} & n\times n\text{ Adjacency matrix for graph }\mathcal{G} \\ & \\ \mathcal{H}\equiv\left( N^{\mathcal{H}},E^{\mathcal{H}} \right) & \text{Graph representing households and family relationships} \\ N^{\mathcal{H}} & \text{Nodes (households) of graph }\mathcal{H} \\ E^{\mathcal{H}} & \text{Ties (family relationships) of graph }\mathcal{H} \\ h & \text{Number of nodes in graph }\mathcal{H} \\ M^{\mathcal{H}} & h\times h\text{ Adjacency matrix for graph }\mathcal{H} \\ & \\ M^{\mathcal{H}\mapsto\mathcal{G}} & n\times h\text{ Matrix mapping individuals to households} \\ N_{i} & \text{Set containing individual }i\text{ as well as individuals connected to }i\text{ in }\mathcal{G} \\ H_{i} & \text{The household of individual }i \\ \Gamma_{i} & \text{Set containing household of individual }i\text{ and connected households in }\mathcal{H} \\ & \\ \mathbf{a} & h\times1\text{ vector of imposed household wealth } \\ \mathbf{Y}\left( \mathbf{a} \right) & n\times1\text{ vector of physical function outcomes under wealth dictated by }\mathbf{a} \\ \mathbf{Y} & n\times1\text{ vector of observed physcial function outcomes} \\ \mathbf{A} & h\times1\text{ vector of observed household wealth} \\ \mathbf{L} & n\times1\text{ vector of observed covariate values} \end{matrix}$$

Unless otherwise indicated, unbolded Latin letters are used to indicate scalar quantities and bold letters indicate vectors. For vector $\mathbf{V}$ and set $A$, $\mathbf{V}_{A}$ is the sub-vector of $\mathbf{V}$ consisting of entries whose indices are included in $A$. Random variables are indicated using capital letters and the realization of a given random variable is indicated using a small letter. (e.g. if $R$ is a random variable, $r$ is a particular value of $R$). Sets are indicated using unbolded capital letters. The set union operator is $\cup$ and the intersection operator is $\cap$. For sets $A$ and $B$, $A\backslash B$ is a set containing all the elements of $A$ except for those contained by $B$.

# Identification Strategy

## Causal Estimands

We assume conditional exchangeability and consistency hold:

$$\mathbf{Y}\left( \mathbf{a} \right)\perp\mathbf{A}|\mathbf{L}\text{ and }\mathbf{Y}=\mathbf{Y}\left( \mathbf{A} \right)$$

where $\mathbf{A}$ is a $h\times1$ vector of observed wealth and $\mathbf{L}$ is a $n\times k$ matrix of covariates.

We are interested in the change in entries of $E\left[ \mathbf{Y}\left( \mathbf{a} \right)|\mathbf{L} \right]$ produced by a unit increase in each entry of $\mathbf{a}$. $E\left[ \mathbf{Y}\left( \mathbf{a} \right)|\mathbf{L} \right]$ is the expected potential outcome for physical function under treatment $a$ for a set of individuals with the same values of covariates $\mathbf{L}$ as were observed. i.e., we are interested in

$$\frac{d}{d\mathbf{a}}E\left[ \mathbf{Y}\left( \mathbf{a} \right)|\mathbf{L} \right]$$

with $ij^{th}$ entry of the form

$$\frac{d}{da_{j}}E\left[ Y_{i}\left( \mathbf{a} \right)|\mathbf{L} \right]$$

Using conditional exchangeability and consistency:

$$\begin{matrix} \frac{d}{d\mathbf{a}}E\left[ \mathbf{Y}\left( \mathbf{a} \right)|\mathbf{L} \right] & =\frac{d}{d\mathbf{a}}E\left[ \mathbf{Y}\left( \mathbf{a} \right)|\mathbf{L},\mathbf{A}=\mathbf{a} \right] \\ & =\frac{d}{d\mathbf{a}}E\left[ \mathbf{Y}|\mathbf{L},\mathbf{A}=\mathbf{a} \right] \end{matrix}$$

We calculate three causal estimands derived from this $n\times h$ matrix — the average direct effect (ADE), average spillover effect (ASE), and average total effect (ATE). ADE is defined as the average change in an individual’s physical function that results from a 1 unit increase in that person’s household’s wealth index. ASE is the average change in an individual’s physical function that results from a 1 unit increase in every other household’s wealth index. The ATE is the sum of ADE and ASE — it is the total average change in individuals’ physical function caused by a 1 unit increase in every household’s wealth index. Formally,

$$\begin{matrix} \text{ATE} & \equiv\frac{1}{n}\sum_{i=1}^{n} \sum_{j=1}^{h} \frac{d}{da_{j}}E[Y_{i}\left| \mathbf{L},\mathbf{A}=\mathbf{a}] \right|_{\mathbf{a}=\mathbf{A}} \\ \text{ASE} & \equiv\frac{1}{n}\sum_{i=1}^{n} \sum_{J\in N^{\mathcal{H}}\backslash\{H_{i}\}} \frac{d}{da_{J}}E[Y_{i}\left| \mathbf{L},\mathbf{A}=\mathbf{a}] \right|_{\mathbf{a}=\mathbf{A}} \\ \text{ADE} & \equiv\frac{1}{n}\sum_{i=1}^{n} \frac{d}{da_{H_{i}}}E[Y_{i}\left| \mathbf{L},\mathbf{A}=\mathbf{a}] \right|_{\mathbf{a}=\mathbf{A}} \end{matrix}$$

## Statistical Model

We assume that $\mathbf{Y}|\mathbf{L},\mathbf{A}$ arises from a conditional Markov random field defined by the following local Markov property Besag (1974):

$$Y_{i}\perp Y_{j}|\mathbf{L}_{N_{i}},\mathbf{A}_{\Gamma_{i}},\mathbf{Y}_{N_{i}\backslash\{i\}}\text{ where }j\notin N_{i}.$$

$\mathbf{L}_{N_{i}}$ is the sub-vector of $\mathbf{L}$ corresponding to entries whose indices are contained in $N_{i}$ . $\mathbf{Y}_{N_{i}\backslash\{i\}}$ and $\mathbf{A}_{\Gamma_{i}}$ are similarly defined.

In addition, we make an assumption about the conditional mean of $\mathbf{Y}$. For ease of presentation, we show the case where $\mathbf{L}$ is a $n\times1$ matrix (as opposed to a $n\times k$ matrix):

$$E\left[ Y_{i}│\mathbf{L},\mathbf{A},\mathbf{Y}_{N^{\mathcal{G}}\backslash\{i\}} \right]=\beta_{0}+\beta_{1}a_{H_{i}}+\beta_{2}l_{i}+\beta_{3}\sum_{j\in\Gamma_{i}\backslash\{H_{i}\}} a_{j}+\sum_{j\in N_{i}\backslash\{i\}} \left( \beta_{4}l_{j}+\phi y_{j} \right) \left( 1 \right)$$

We refer to the above equation as the conditional mean model. Through a slight abuse of notation, we can re-write this as:

$$E\left[ \mathbf{Y}│\mathbf{L},\mathbf{A},\mathbf{Y} \right]=\beta_{0}\mathbf{1}_{n}+\beta_{1}M^{\mathcal{H}\mapsto\mathcal{G}}\mathbf{A}+\beta_{2}\mathbf{L}+\beta_{3}M^{\mathcal{H}\mapsto\mathcal{G}}M^{\mathcal{H}}\mathbf{A}+\beta_{4}M^{\mathcal{G}}\mathbf{L}+\phi M^{\mathcal{G}}\mathbf{Y}$$

Now:

$$\begin{matrix} E\left[ E\left[ \mathbf{Y}│\mathbf{L},\mathbf{A},\mathbf{Y} \right]|\mathbf{L},\mathbf{A} \right] & =\beta_{0}\mathbf{1}_{n}+\beta_{1}M^{\mathcal{H}\mapsto\mathcal{G}}\mathbf{A}+\beta_{2}\mathbf{L}+\beta_{3}M^{\mathcal{H}\mapsto\mathcal{G}}M^{\mathcal{H}}\mathbf{A}+\beta_{4}M^{\mathcal{G}}\mathbf{L}+\phi M^{\mathcal{G}}E\left[ \mathbf{Y}│\mathbf{L},\mathbf{A} \right] \\ E\left[ \mathbf{Y}│\mathbf{L},\mathbf{A} \right] & =\left( \mathbf{I}_{n}-\phi M^{\mathcal{G}} \right)^{-1}\left( \beta_{1}M^{\mathcal{H}\mapsto\mathcal{G}}+\beta_{3}M^{\mathcal{H}\mapsto\mathcal{G}}M^{\mathcal{H}} \right)\mathbf{A}+... \end{matrix}$$

We can write an $n\times h$ Jacobian matrix:

$$\frac{d}{d\mathbf{a}}E\left[ \mathbf{Y}│\mathbf{A}=\mathbf{a},\mathbf{L} \right]=\left( \mathbf{I}_{n}-\phi M^{\mathcal{G}} \right)^{-1}\left( \beta_{1}M^{\mathcal{H}\mapsto\mathcal{G}}+\beta_{3}M^{\mathcal{H}\mapsto\mathcal{G}}M^{\mathcal{H}} \right)$$

and using this matrix, calculate ADE, ASE and ATE as:

$$\begin{matrix} ATE & =\frac{1}{n}\text{grandsum}\{\left( \mathbf{I}_{n}-\phi M^{\mathcal{G}} \right)^{-1}\left( \beta_{1}M^{\mathcal{H}\mapsto\mathcal{G}}+\beta_{3}M^{\mathcal{H}\mapsto\mathcal{G}}M^{\mathcal{H}} \right)\} \\ ADE & =\frac{1}{n}\text{trace}\{M^{\mathcal{H}\mapsto\mathcal{G}}'\left( \mathbf{I}_{n}-\phi M^{\mathcal{G}} \right)^{-1}\left( \beta_{1}M^{\mathcal{H}\mapsto\mathcal{G}}+\beta_{3}M^{\mathcal{H}\mapsto\mathcal{G}}M^{\mathcal{H}} \right)\} \\ ASE & =ATE-ADE \end{matrix}$$

Where $\text{grandsum}\{A\}$ is the sum of all the entries in matrix $A$.

# Estimation

## Estimation with Complete Data

Estimation of the causal estimands proceeded in two steps. First we estimated the coefficients of the conditional mean model ([Equation 1](#eq-mean-model)). Following that, we calculated the causal estimates, ATE, ADE, and ASE, which are each a deterministic function of the coefficients.

**Box 2 (Finding a Stable Set)** Following Tchetgen Tchetgen et al. (2020), we define a stable set of $\mathcal{G}\equiv\left( N^{\mathcal{G}},E^{\mathcal{G}} \right)$ as graph $\mathcal{G}^{\left[ S \right]}\equiv\left( N^{\left[ \mathcal{G} \right]},E^{\left[ \mathcal{G} \right]} \right)$ such that $N^{\left[ \mathcal{G} \right]}\subset N^{\mathcal{G}}$ and $E^{\mathcal{G}}=\emptyset$.

We used the following algorithm to compute stable sets:

1. Set $A\leftarrow N^{\mathcal{G}}$ and $B\leftarrow\emptyset$.
2. Include all singletons of $\mathcal{G}$ in $B$. i.e., $B\leftarrow\{i\in A:N_{i}=\{i\}\}$
3. Repeat the following steps until $A=\emptyset$
   1. Randomly select $k\in A$
   2. Include $k$ in $B$: $B\leftarrow B\cup\{k\}$
   3. Remove $N_{k}$ from $A$: $A\leftarrow A\backslash N_{k}$

Then $\mathcal{G}^{\left[ S \right]}=\left( N^{\left[ \mathcal{G} \right]},\emptyset\right)$ where $N^{\left[ \mathcal{G} \right]}=A$

### Conditional Mean Model

Estimating the parameters of the conditional mean model was complicated by the fact that the observations belonging to a pair of individuals who are connected in the network are possibly correlated with each other. Proceeding as if the observations are independent may lead to biased estimates. To account for correlation among observations, we use the coding estimator described in Tchetgen Tchetgen et al. (2020).

Briefly, we find a stable set (see [Box 2](#thm-stable-set) ) — a sub-network of $\mathcal{G}$ such that no two individuals in the sub-network are connected in $\mathcal{G}$ and such that each individual in the sub-network is a member of HAALSI. (The latter restriction was made because those are the only individuals for which we have a measure of the outcome). Because of the local Markov assumption, this subset consists of conditionally independent observations, given their own treatments and covariates, and their family members’ treatments, covariates, and outcomes.

We then estimate parameters of the conditional mean model using standard generalized estimating equations with robust standard errors.

The coding estimator can be inefficient if outcomes among connected units are truly uncorrelated after conditioning on their individual and network exposures and covariates — an assumption we test empirically. Under a sub-model of the conditional mean model where $\phi=0$, each individual’s physical function outcome is mean-independent of other individuals’ physical function outcomes. If there were no empirical evidence against such hypothesized sub-model, it would be reasonable to assume that outcomes of directly connected units are independent, so that one can estimate the parameters of the mean model using a standard linear regression for independent outcomes, fitted using all available data. In this sub-model, we would not have to account for correlation between observations meaning that we would not need to use a stable-set.

We conducted a Wald hypothesis test (using robust standard errors), assessing whether $\phi=0$. This was done by fitting the linear regression model implied by the conditional mean model ([Equation 1](#eq-mean-model)) while using all HAALSI data. This model is correct under the null hypothesis that $\phi=0$ conditional on covariates. Where the null hypothesis was not rejected, we assumed that the data arise from the sub-model defined by conditional independence among observed outcomes.

### Causal Parameters

As shown above, the causal estimates of interest are calculated as a deterministic function of the coefficients of the conditional mean model, household membership, age, and the structure of the family network.

We used the parametric bootstrap to conduct statistical inference for these quantities. We simulated 5000 realizations from the asymptotic joint normal distribution of the regression coefficients of the conditional mean model, each time calculating ADE, ASE, and ATE. We took the $0.025$ and $0.975$th quantiles of the empirical distributions as the bounds of the $95$ confidence interval.

## Estimation with Missingness

### Dataset

Covering a $420$ $km^{2}$ region of Mpumalanga Province, South Africa, Agincourt Health and Demographic Surveillance System (AHDSS) has conducted an annual survey of households, collecting information on births, deaths, migrations, and family relationships since 1992. In addition, AHDSS fieldworkers collected information on household wealth once every two years beginning in 2001 and annually since 2013 (Kabudula et al., 2017; Kahn et al., 2012). The Health and Aging in Africa: A Longitudinal Study of an INDEPTH Community in South Africa (HAALSI) is a longitudinal cohort or older adults nested in the AHDSS. The HAALSI cohort is comprised of 5059 adults who were sampled from residents of AHDSS who were over 40 years of age in 2014 (Gómez-Olivé et al., 2018).

We used AHDSS data on household wealth as the main exposure and HAALSI data on disability and physical function as the main outcome.

Some individuals were missing data on household wealth, inducing missingness in network wealth. In addition, physical function was only measured among HAALSI participants and not among all AHDSS residents. This meant that among HAALSI participants, network physical function measures are not possible to compute directly. If we attempted to compute network physical function by summing over only the valid values of physical function among each respondent’s direct ties, they would be right-censored. To account for missingness, we conducted two separate sets of analyses. The main analysis is based on multiple imputation using chained equations. This analysis is predicated on the assumption that missingness depends only on observed variables (Rubin, 1976). The secondary analysis dropped entries that were missing the outcome value and mean-imputed household wealth. Results from this analysis are unbiased under the unrealistic assumption that data are missing completely at random (Rubin, 1976).

#### Measures

##### Social Network

To quantify the resources held in family networks, we constructed a sociocentric family network among all individuals in the AHDSS. In this network, nodes represent individuals and ties represent their relationships with first- or second-degree relatives. First-degree relatives are defined as parents, children, and conjugal partners and second-degree relatives are the first-degree relatives of first-degree relatives. For a given individual, we use the term ‘family members’ to mean the group consisting of first- and second-degree relatives.

We used a sub-network consisting only of members of the HAALSI cohort along with their family members regardless of whether they were themselves in the HAALSI cohort or not. We call this the HAALSI community network (HCNet).

##### Household and Network Wealth

Household wealth was assessed using a DHS asset index that measure that incorporates information on household infrastructure and goods (Payne et al., 2017; Rutstein et al., 2004). Measurements of household wealth were made every two years from 2001 to 2013. For each year in this period, each individual in the HCNet was associated with the measure of household wealth that was recorded for her household. Network wealth was calculated as the sum of household wealth among the households of family members. Where two or more family members lived in the same household, that household’s wealth contributed only once to network wealth.

##### Individual and Network Physical Functioning

In HAALSI, individual grip strength was measured using a Smedley digital hand dynamometer, taking two measurements per hand. Following Payne et al. (2017), we used the average of the grip strength measures on the participant’s self-reported dominant hand. For participants who reported being ambidextrous, we took the average of the two highest measures regardless of which hand they were measured on. Measures above $75$ $kg$ were treated as out of range and therefore missing. An individual’s network grip strength was measured as the sum of grip strength values among her family members who were over $40$ years of age in $2014$.

##### Individual and Network Gait Speed

Individual gait speed was measured among HAALSI participants using a timed walk. Interviewers marked a length of $2.5$ meters on an obstacle-free floor. The respondent was asked to walk from one end to the other, and she was timed. The respondent was then asked to turn around and return to the point of origin while being timed. Gait speed was calculated by dividing 5 by the sum of the times (in seconds). Gait speeds below $0.2$ $m/s$ or above $2$ $m/s$ were treated as out of range and therefore missing. Network gait speed was measured as the sum of gait speed values among family members who were over $40$ years of age in $2014$.

##### Individual and Network Activities of Daily Living

Limitations in activities of daily living (ADL) was measured in HAALSI using a set of questions asking whether the respondent is unable, or finds it difficult, to bathe, eat, get out of bed, toilet, or walk across the room unaided. Individual ADL is equal to $1$ if the individual had at least one limitation and $0$ otherwise. Network ADL is the sum of ADL values among each individual’s family members who were above $40$ years of age in $2014$.

### Mean-Imputation (Secondary) Analysis

In this analysis, an individual was excluded from the analysis if she was missing the physical function outcome, or household wealth, or if all the households in which her family members live were missing household wealth, or if all connected individuals were missing the physical function outcome.

If at least one connected household had valid household wealth, then network wealth was calculated by summing the valid values of household wealth and up-weighting the sum by the ratio of connected households to valid household wealth measurements.

i.e. we computed network wealth as:

$$\frac{\text{\# Connected Households}}{\text{\# Valid HH Wealth Measures}}\times\text{sum of valid HH Wealth Measures}$$

Similarly, if at least one connected individual had valid physical function, we calculated network physical function by summing the valid values of physical function and up-weighting the sum by the ratio of # connected individuals to # valid physical function values

i.e. we computed network physical function as:

$$\frac{\text{\# Family Members}}{\text{\# Valid Phys Func Meausres}}\times\text{sum of valid Phys Func Measures}$$

### Multiple Imputation using Chained Equations

We imputed missing values of household wealth and physical function using a custom-built R package based on the multiple imputation using chained equations (MICE) algorithm Van Buuren et al. (2006). In each iteration of this algorithm, we imputed missing household wealth values using household-level measures as predictors and calculated network wealth values using these imputed values.

[Table 1](#tbl-imputation-ind-missing) shows the level of missingness in household and network wealth for two sub-networks: the HAALSI network consists of individuals in the HAALSI cohort. The Community network consists of individuals in HAALSI as well as individuals who are connected to those in HAALSI. In addition to household and network wealth, the following Level 2 variables were used in imputation model (these variables were fully observed):

For each year: Number of People in Household, Number of Males > 60 years of age, Number of Females > 60 years of age, Number of Males 18-60 years of age, Number of Children Network Degree

In the same iteration of the algorithm, we then imputed individual level physical function using individual level measures in addition to household-level measures as predictors, and calculated network physical function. Table 3 shows the level of missingness for in household and network physical function for the same sub-networks described above. In addition to Level-2 variables, individual physical function, and network physical function, we used individual network degree to impute physical function.

We iterated these steps 60 times for each imputed dataset, creating 64 such datasets. Dichotomous variables were imputed using a random draw from the Bernoulli distribution with probability of success given by predicted values from a logistic regression. Continuous variables were imputed using predictive mean matching, choosing randomly from the 5 closest predicted values.

Table 1: Level-2 Imputation: Individuals

|  | Individual Attributes | | Network Attributes | |
| --- | --- | --- | --- | --- |
|  | % Missing HAALSI | % Missing Community | % Missing HAALSI | % Missing Community |
| ADL (W1) | 0.2 | 60.6 | 46.2 | 34.5 |
| ADL (W2) | 17.8 | 67.6 | 54.3 | 45.2 |
| Grip Strength (W1) | 9.6 | 64.4 | 48.6 | 39.2 |
| Grip Strength (W2) | 36.4 | 74.9 | 58.9 | 54.2 |
| Gait Speed (W1) | 8.1 | 63.8 | 48.0 | 38.5 |
| Gait Speed (W2) | 39.3 | 76.0 | 59.4 | 55.9 |
| Education at Baseline | 0.3 | 60.7 | NA | NA |
| Employment at Baseline | 0.3 | 60.6 | NA | NA |
| Married at Baseline | 0.1 | 60.6 | NA | NA |
| Has Children at Baseline | 0.1 | 60.6 | NA | NA |
| Recieves Pension Income at Baseline | 0.0 | 60.5 | NA | NA |
| Wealth 2001 | 6.3 | 6.8 | 30.8 | 27.7 |
| Wealth 2003 | 3.7 | 3.8 | 29.3 | 26.6 |
| Wealth 2005 | 6.6 | 7.0 | 29.4 | 26.3 |
| Wealth 2007 | 9.0 | 9.5 | 30.9 | 28.5 |
| Wealth 2009 | 4.1 | 4.3 | 28.2 | 25.8 |
| Wealth 2011 | 7.9 | 8.9 | 29.3 | 26.5 |
| Wealth 2013 | 8.4 | 10.8 | 29.8 | 27.0 |
| Wealth 2014 | 5.3 | 7.6 | 27.8 | 25.0 |
| Wealth 2015 | 7.3 | 9.3 | 27.5 | 25.0 |
| Wealth 2016 | 8.2 | 10.1 | 27.7 | 25.1 |
| Wealth 2017 | 17.6 | 20.7 | 33.5 | 30.7 |

Table 2: Level-2 Imputation: Households

|  | Household Attributes | | Network Attributes | |
| --- | --- | --- | --- | --- |
|  | % Missing HAALSI | % Missing Community | % Missing HAALSI | % Missing Community |
| Wealth 2001 | 6.6 | 9.2 | 31.3 | 20.7 |
| Wealth 2003 | 3.9 | 6.0 | 29.9 | 19.1 |
| Wealth 2005 | 7.0 | 10.2 | 30.0 | 18.8 |
| Wealth 2007 | 9.6 | 13.0 | 31.5 | 20.1 |
| Wealth 2009 | 4.4 | 7.1 | 28.6 | 16.8 |
| Wealth 2011 | 8.3 | 14.8 | 29.6 | 17.1 |
| Wealth 2013 | 9.0 | 18.6 | 30.2 | 17.2 |
| Wealth 2014 | 5.7 | 13.9 | 28.0 | 15.2 |
| Wealth 2015 | 7.7 | 16.5 | 27.8 | 15.4 |
| Wealth 2016 | 8.9 | 17.2 | 27.9 | 15.4 |
| Wealth 2017 | 18.5 | 30.0 | 33.5 | 20.5 |

# Results

## Conditional Mean Model

In [Figure 1](#fig-cond-adl-sup), [Figure 2](#fig-cond-grip-sup), and [Figure 3](#fig-cond-gait-sup), we show results from the conditional mean models for ADL, grip strength, and gait speed, respectively. Since we did not reject the hypothesis that $\phi=0$ for gait speed and ADL, we concluded that for these outcome measures, observations were conditionally independent. As a result, we fitted regression models for these outcomes using the entire HCNet dataset. By contrast, there was evidence of positive conditional network dependence of grip strength among directly connected individuals, as [Figure 2](#fig-cond-grip-sup) shows.

For all three outcomes q and years t, we failed to reject the null hypothesis that $\beta_{3,t,q}=0$ at type 1 error level of 0.05. We conclude that after accounting for an individual’s household wealth, there is no evidence that the wealth of family members’ households is associated with her physical function or disability outcome.

Finally, we found weak evidence of a negative association between household wealth and ADL, and weak evidence of a negative association between household wealth and Gait Speed. That is, greater household wealth predicted slower gait speed and fewer limitations. In both cases, multiple imputation results showed a stronger negative association than mean imputation results. In both imputation analyses, we found relatively strong evidence of a positive association between household wealth and grip strength.

| 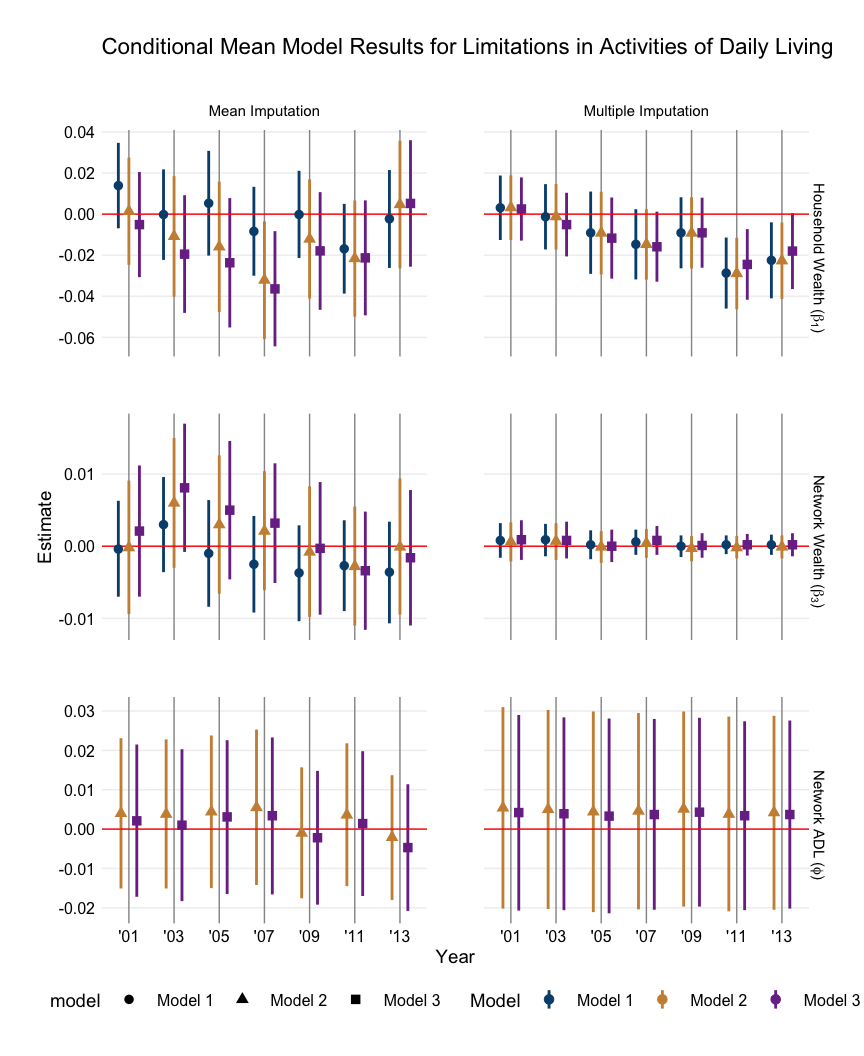  Figure 1: Conditional Mean Model Results for Activities of Daily Living Limitations (ADL). Model 1 includes Network Wealth and Household Wealth as predictors. Model 2 includes these as well as Network ADL as predictors. Model 3 includes these as predictors and adjusts age and gender as potential confounders. The top row shows results from the mean imputation analysis and the bottom row shows results from the multiple imputation analysis. The first column shows the point estimate and confidence interval for household wealth ($\beta_{1,t,q}$), the second column shows results for network wealth ($\beta_{3,t,q}$), and the final column shows the coefficient for the network value of physical function ($\phi_{2014,q}$) |
| --- |

| 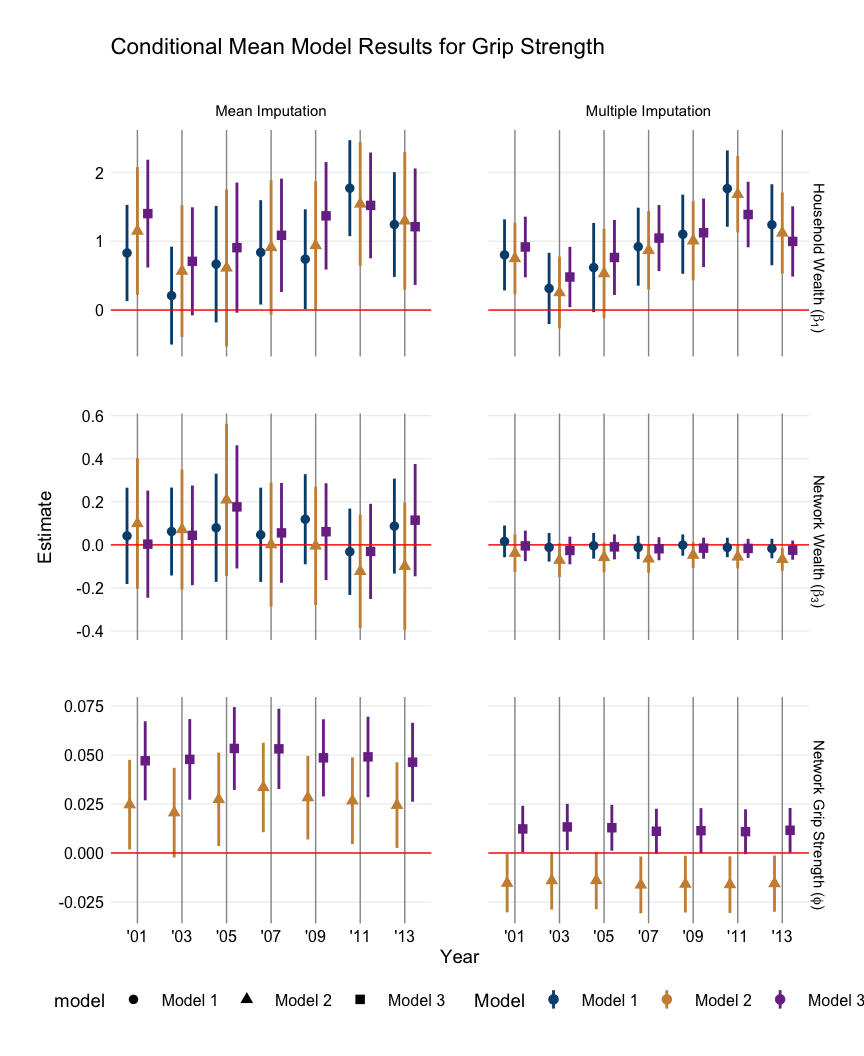  Figure 2: Conditional Mean Model Results for Grip Strength. Model 1 includes Network Wealth and Household Wealth as predictors. Model 2 includes these as well as Network Grip Strength as predictors. Model 3 includes these as predictors and adjusts age and gender as potential confounders. The top row shows results from the mean imputation analysis and the bottom row shows results from the multiple imputation analysis. The first column shows the point estimate and confidence interval for household wealth ($\beta_{1,t,q}$), the second column shows results for network wealth ($\beta_{3,t,q}$), and the final column shows the coefficient for the network value of physical function ($\phi_{2014,q}$) |
| --- |

| 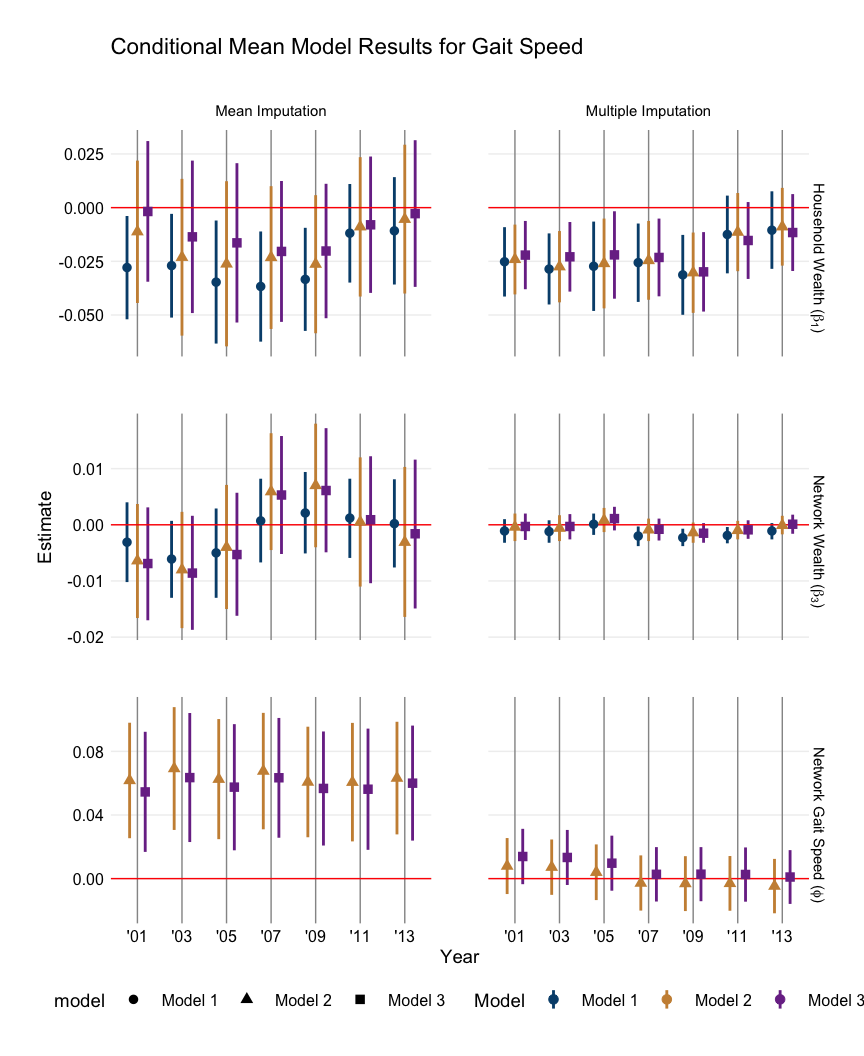  Figure 3: Conditional Mean Model Results for Gait Speed. Model 1 includes Network Wealth and Household Wealth as predictors. Model 2 includes these as well as Network Gait Speed as predictors. Model 3 includes these as predictors and adjusts age and gender as potential confounders. The top row shows results from the mean imputation analysis and the bottom row shows results from the multiple imputation analysis. The first column shows the point estimate and confidence interval for household wealth ($\beta_{1,t,q}$), the second column shows results for network wealth ($\beta_{3,t,q}$), and the final column shows the coefficient for the network value of physical function ($\phi_{2014,q}$) |
| --- |

## Causal Parameters

Since for gait speed and for ADL, network wealth and network physical function were not associated with the outcome, we can conclude that the average spillover effect was 0 for both these outcomes. [Figure 4](#fig-estimands-grip-sup) displays the causal estimates for the effect of household wealth on grip strength. In the mean imputation analysis, there was no statistical evidence that the average direct effect or average spillover effect were different from zero for gait speed. In the multiple imputation analysis, there was some statistical evidence of a negative average direct effect of household wealth on gait speed.

| 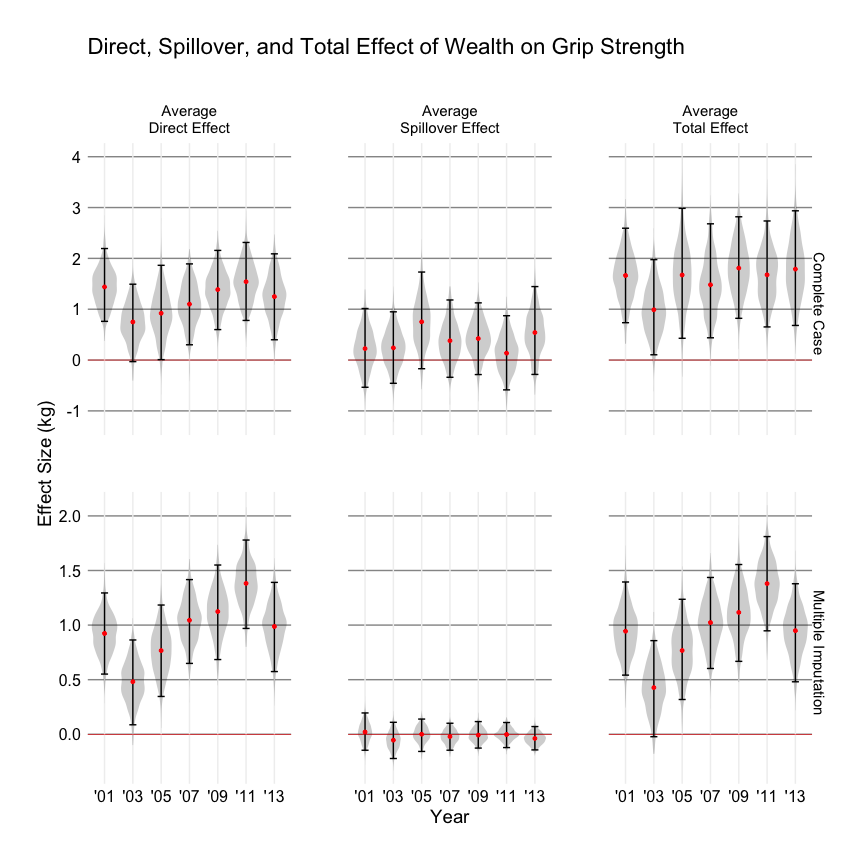  Figure 4: Causal Estimates of the Effect of Wealth on Grip Strength. The top row shows results from the mean imputation analysis and the bottom row shows results from the multiple imputation analysis. The first column shows the point estimate and confidence interval for ADE, the second column shows results for ASE, and the final column shows results for ATE. |
| --- |

# References

Besag, J. (1974). Spatial interaction and the statistical analysis of lattice systems. *Journal of the Royal Statistical Society: Series B (Methodological)*, *36*(2), 192225.

Gómez-Olivé, F. X., Montana, L., Wagner, R. G., Kabudula, C. W., Rohr, J. K., Kahn, K., Bärnighausen, T., Collinson, M., Canning, D., Gaziano, T., Salomon, J. A., Payne, C. F., Wade, A., Tollman, S. M., & Berkman, L. (2018). Cohort Profile: Health and Ageing in Africa: A Longitudinal Study of an INDEPTH Community in South Africa (HAALSI). *International Journal of Epidemiology*, *47*(3), 689–690j. <https://doi.org/10.1093/ije/dyx247>

Kabudula, C. W., Houle, B., Collinson, M. A., Kahn, K., Gómez-Olivé, F. X., Tollman, S., & Clark, S. J. (2017). Socioeconomic differences in mortality in the antiretroviral therapy era in Agincourt, rural South Africa, 2001-13: a population surveillance analysis. *The Lancet. Global Health*, *5*(9), e924–e935. <https://doi.org/10.1016/S2214-109X(17)30297-8>

Kahn, K., Collinson, M. A., Gómez-Olivé, F. X., Mokoena, O., Twine, R., Mee, P., Afolabi, S. A., Clark, B. D., Kabudula, C. W., Khosa, A., Khoza, S., Shabangu, M. G., Silaule, B., Tibane, J. B., Wagner, R. G., Garenne, M. L., Clark, S. J., & Tollman, S. M. (2012). Profile: Agincourt health and socio-demographic surveillance system. *International Journal of Epidemiology*, *41*(4), 988–1001. <https://doi.org/10.1093/ije/dys115>

Payne, C. F., Wade, A., Kabudula, C. W., Davies, J. I., Chang, A. Y., Gomez-Olive, F. X., Kahn, K., Berkman, L. F., Tollman, S. M., Salomon, J. A., & Witham, M. D. (2017). Prevalence and correlates of frailty in an older rural African population: findings from the HAALSI cohort study. *BMC Geriatrics*, *17*(1). <https://doi.org/10.1186/s12877-017-0694-y>

Rubin, D. B. (1976). Inference and missing data. *Biometrika*, *63*(3), 581–592. <https://doi.org/10.1093/biomet/63.3.581>

Rutstein, S. O., Johnson, K., MEASURE, O. M., & others. (2004). *The DHS wealth index*. ORC Macro, MEASURE DHS.

Tchetgen Tchetgen, E. J., Fulcher, I. R., & Shpitser, I. (2020). Auto-g-computation of causal effects on a network. *Journal of the American Statistical Association*, 112.

Van Buuren, S., Brand, J. P., Groothuis-Oudshoorn, C. G., & Rubin, D. B. (2006). Fully conditional specification in multivariate imputation. *Journal of Statistical Computation and Simulation*, *76*(12), 10491064.
